# Insect-inspired breathing interfaces: investigating robustness of coating-free gas entrapping microtextured surfaces under pressure cycles

**DOI:** 10.1038/s44172-024-00231-2

**Published:** 2024-06-21

**Authors:** Sankara Arunachalam, Muhammad Subkhi Sadullah, Himanshu Mishra

**Affiliations:** 1 Environmental Science and Engineering Program, Biological and Environmental Sciences and Engineering Division, King Abdullah University of Science and Technology (KAUST), Thuwal, 23955-6900 Kingdom of Saudi Arabia; 2 Water Desalination and Reuse Center, King Abdullah University of Science and Technology, Thuwal, Kingdom of Saudi Arabia; 3 Center for Desert Agriculture, King Abdullah University of Science and Technology, Thuwal, Kingdom of Saudi Arabia

**Keywords:** Mechanical engineering, Fluidics

## Abstract

Numerous natural and engineering scenarios necessitate the entrapment of air pockets or bubbles on submerged surfaces. Current technologies for bubble entrapment rely on perfluorocarbon coatings, limiting their sustainability. Herein, we investigated the efficacy of doubly reentrant cavity architecture towards realizing gas-entrapping microtextured surfaces under static and dynamic pressure cycling. The effects of positive (>1 atm), negative (<1 atm), and positive–negative cycles on the stability the gas entrapment inside individual doubly reentrant cavities were studied across a range of pressures, ramp rates, intercycle intervals, and water-column heights. Remarkably, the fate of the trapped air under pressure cycling fell into either of the following regimes: the bubble (i) monotonically depleted (unstable), (ii) remained indefinitely stable (stable), or (iii) started growing (bubble growth). This hitherto unrealized richness of underwater bubble dynamics should guide the development of coating-free technologies and help us understand the curious lives of air-breathing aquatic and marine insects.

## Abstract (web summary)

Sankara Arunachalam and colleagues explore the effects of cyclic pressure on the fate of air trapped inside microtextured surfaces submerged in water. The findings guide the design and function of gas-entrapping microtextured surfaces and offer insights into survival strategies of underwater breathers.

## Introduction

When air-breathing animals venture underwater, the robustness of the air entrapped on/around their bodies is a matter of life and death. Broadly, air entrapment occurs in the form of compressible bubbles or incompressible “plastron” (i.e., physical gill)^1^. Examples of air-breathers exploring the underwater realm include the aquatic beetle (*Potamodytes tuberosus*), which feeds on microbial slime under shallow rock surfaces in flowing waters^2^; sea-skaters (e.g., *Halobates germanus*, *Halobates melleus*, and *Halobates hayanus*), which may accidentally drown after being hit by waves or rain drop and yet survive underwater for over 18 h^3–5^; and the semiaquatic *Anolis* lizard species (e.g., *Anolis eugenegrahami* and *Anolis aquaticus*), which can respire for up to 18 min underwater^6^. These animals have evolved strategies for combining their water-repellent secretions with specialized hair or skin patterns to sustain underwater respiration^5–14^. Animal behavior under these life-threatening scenarios is fascinating but poorly understood. In addition, there is a growing expectation for their biomimicry to unleash sustainable technologies for frictional drag reduction^15–17^, fouling-resistant membranes in fermenter broths^18^, mitigating cavitation damage^19^, packaging electronics^20,21^, oxygen extraction from water^22,23^, food–water security^24,25^, and breathing textiles^26^.

**Fig. 1:**
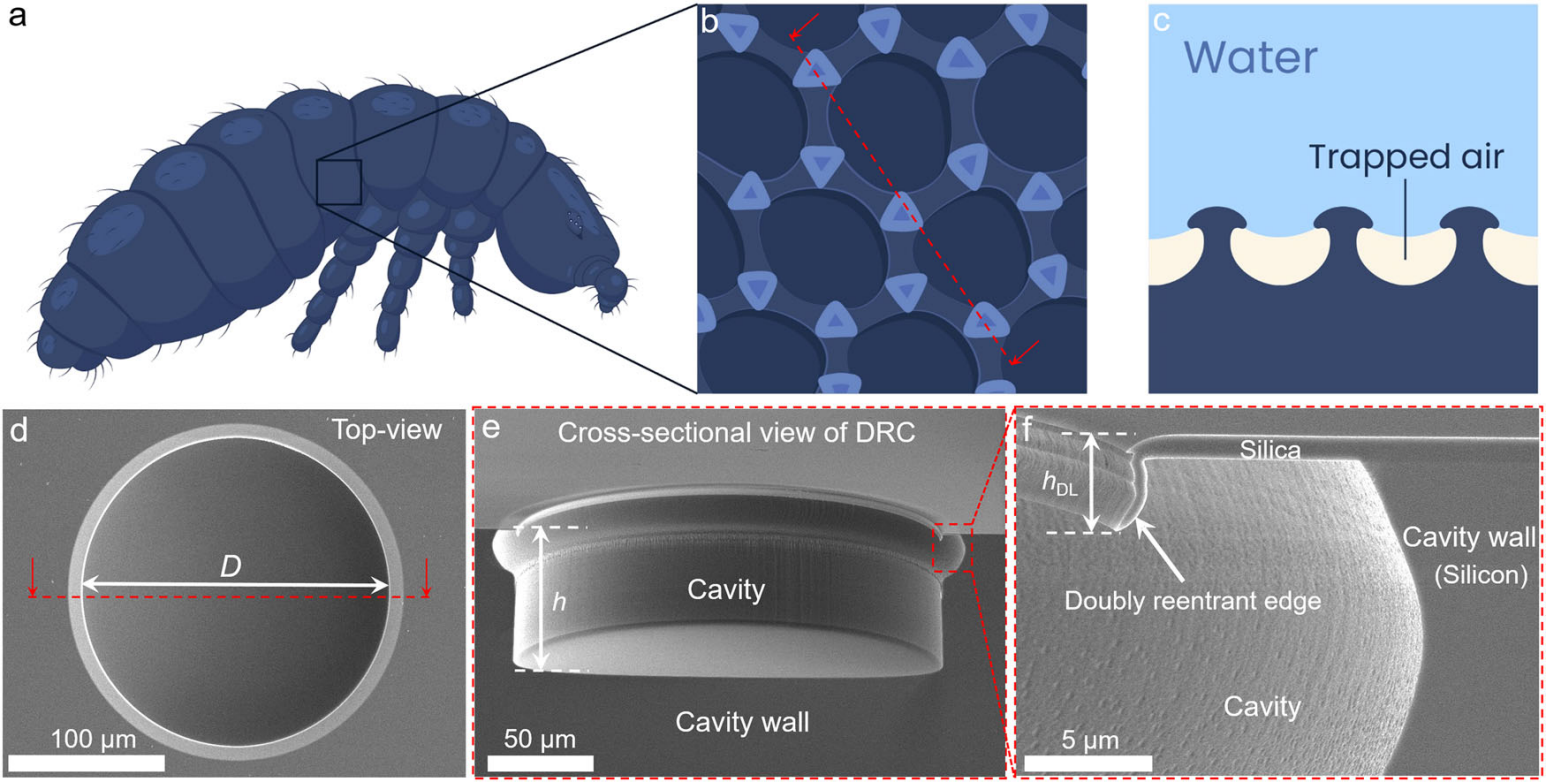
Illustration of springtail cuticles and representative scanning electron microscopy (SEM) images of doubly reentrant cavities (DRCs). **a**–**c** Schematics of springtail cuticles (Collembola) with mushroom-shaped overhanging features that enable them to entrap air upon water immersion. Artistic reconstruction of cuticles and its cross-sectional view along the dotted lines in Fig. 1b inspired by Supplementary Fig. 1 and images presented previously^28^. **d**–**f** Representative SEM image of the top view, cross-sectional view along the dotted lines in Fig. 1d, and magnified view of DRCs with diameter *D* = 200 µm and depth *h* ≈ 67 µm.

We are quite impressed by springtails (Collembola), which are ubiquitous arthropods that live in moist soil where flooding is frequent (Fig. 1a–c and Supplementary Fig. 1). They breathe through their exoskeleton, which features cavities that have mushroom-shaped overhangs at their inlets, commonly known as doubly reentrant cavities (DRCs)^10,27–30^. The mushroom-shaped (or doubly reentrant) features, could stabilize the intruding menisci of wetting liquids in ‘metastable states’ by trapping air in the microtexture, regardless of the wettability of the material. The DRC architecture has been extensively investigated for enhancing boiling nucleation^31–34^. In their pioneering contribution, Kim et al.^32^ inverted the DRC architecture to realize a doubly reentrant pillar (DRP) microtexture that can repel droplets of wetting liquids with surface tension as low as 10 mN/m (placed from the top)^16^. Interestingly, air-filled (or the Cassie states)^35^ DRP arrays may catastrophically transition to the fully filled (or the Wenzel state)^36^ if the wetting liquid approaches laterally (e.g., at the array boundary or in the presence of localized defects/damage). The DRC architecture does not undergo such catastrophic wetting transitions because of its compartmentalized nature^28,37–44^. In fact, we put forth the concept of gas-entrapping microtextured surfaces (GEMS) comprised of arrays of DRCs that when submerged under hexadecane, entrapped air for over a month while the intrinsic contact angle was *θ*_o_ = 20°^19,39,45^. In contrast, simple cylindrical cavities of the same size and chemical makeup were filled in ~1 s. This >10^7^-time delay in wetting transitions underscores the efficacy of the DRC architecture toward realizing coating-free liquid repellence, which is desirable because of the adverse effects of perfluorinated chemicals^46,47^. Proof-of-concept demonstrations of DRC-based GEMS^45^ and gas-entrapping membranes^48,49^ produced using low-cost wetting materials suggest that it may be possible to develop perfluorocarbon-free green technologies.

As a criterion for assessment, researchers have studied the stability of air-filled states of DRCs under hydrostatic columns of (wetting) liquids as a function of the liquid’s vapor pressure, capillary condensation, apparent contact angles, and gas diffusion^39,50^. However, to fully assess the pros and cons of the GEMS architecture, it is crucial to challenge them against pressure fluctuations, which are common in practical scenarios, such as pipes and fittings^51^, cavitation in pumps^52,53^, microfluidic devices^54,55^, pulse flows for cleaning membrane^56^, and oscillating bubble–induced jet flow for cleaning surfaces^57^. How the DRC air entrapment would fare under cyclic pressure under water, i.e., the longevity of Cassie-to-Wenzel transitions, is largely unknown and unexplored.

In this study, we submerged GEMS under varying depths of water columns and investigated the fate of the entrapped air on the pressurization/depressurization rates, time intervals between cycles, and gas saturation level of the water. A broad spectrum of techniques was used to address the following interrelated elemental questions: (i) how does the breakthrough pressure (BtP) vary with the pressurization rate; (ii) how does the trapped air “bubbles” respond to cyclic pressure of positive (>1 atm), negative (<1 atm), and positive–negative triangular waves; (iii) what are the effects of the time interval between cycles on the bubble stability and DRC filling time; and (iv) how do wetting transitions depend on the water column height and gas saturation level. Notably, our findings revealed that using DRCs, coating-free microtextured surfaces that can trap air indefinitely under cyclic pressure can be realized with the judicious selection of the cavity size, water column height, and time interval between cycles.

## Results

In this study, GEMS comprised of a single DRC that was realized onto commercial SiO_2_/Si wafers via microfabrication protocols that we have reported recently^37–39,45^ (Fig. 1d–f; refer to Methods for details). Briefly, photolithography, dry etching of SiO_2_ and Si layers, and thermal oxide were exploited to microfabricate DRCs with diameter *D* = 200 µm and two depths: *h* ≈ 55 and 67 µm. We realized a single DRC per ~1 × 1 cm^2^ of SiO_2_/Si wafer substrate instead of an array because neighboring air bubbles in water^58,59^ or evaporating droplets in air^60^ are known to exchange gas (or vapor) with each other and cause interference when determining lifetime. Therefore, our samples with single DRCs obviated such interference during wetting transitions.

Because the typical sample investigated in this work was ~1 × 1 cm^2^ in size with a DRC with a 200-µm diameter in the middle, most of the sample surface was smooth and flat. Wetting of the flat silica surface was first characterized with 6-µL water droplets by advancing and retracting them at ± 0.2 µL/s. The advancing and receding apparent contact angles were *θ*_A_ = 65° ± 2° and *θ*_R_ = 30° ± 2°. However, standard contact angle goniometry could not be applied to characterize the wetting of individual DRCs of size *D* = 200 µm and depth *h* ≈ 67 µm. In response, we applied the newest criterion for characterizing the omniphobicity of surfaces via immersion^37^. Given the hydrophilicity of silica, thermodynamics mandates that the DRC–water–air system must reside in the fully filled (or the Wenzel) state^36,41^. However, as the sample was submerged under a 5-mm-high water column, the DRC entrapped air inside it, and the subsequent Cassie-to-Wenzel wetting transition took >13 days (Supplementary Fig. 2). This result underscores the efficacy of this coating-free approach under hydrostatic scenarios. We chose water (surface tension, ${\gamma }_{{{{{{\rm{LV}}}}}}}$ = 72 mN m^−1^ and viscosity, *μ* = 1 mPa.s at standard temperature and pressure conditions) as the probe liquid because of its ease of use and availability.

### BtP and effect of the ramp rate

**Fig. 2:**
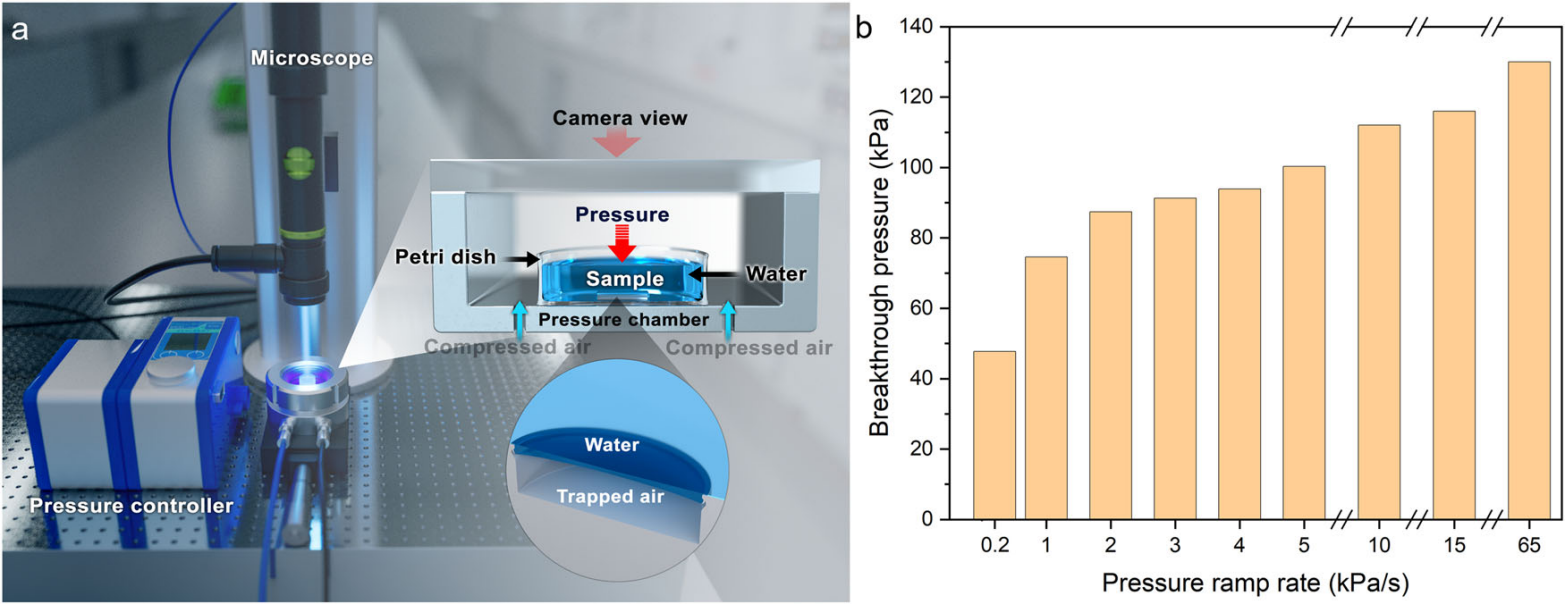
Experimental setup and influence of pressurization rate on BtP. **a** Schematic of the custom-built cell for studying the effects of static and cyclic pressure on a single doubly reentrant cavity **(**DRC). Headspace above the submerged sample is pressurized/depressurized, and wetting transitions are recorded using an overhead optical microscope. **b** BtP of DRC as a function of pressure ramp rate; BtP is defined as the pressure at which intruding water meniscus touches the cavity floor while being pinned at the DRC edge. The results showed that the higher the ramp rate, the higher the BtP. Note that the DRC’s diameter was 200 µm, and its depth was 55 µm.

Next, the mechanical stability of the air entrapped inside the submerged DRCs was challenged under various hydrostatic pressurization and depressurization scenarios. We built a customized pressure cell that facilitated the samples’ submergence under water columns of known heights while the headspace was pressurized at rates within the following range: 0.2–65 kPa/s (Fig. 2a and Supplementary Fig. 3; Methods). The cell was fitted with a white light microscope attached to a camera to record wetting transitions from the top (Methods).

We defined BtP for DRC as the external hydrostatic pressure at which water meniscus, pinned at the DRC edge, droops inside and touches the cavity floor—the cavity “fails” (Supplementary Fig. 4 and Supplementary Video 1). In this work, cavity “failure” is not the same as cavity “filling,” as the latter is ascribed to a fully water-filled (Wenzel) state. Microtexture BtP is a key descriptor of its robustness against liquid intrusion. Curiously, BtP for 3-mm-thick water columns increased with the ramp rate within the abovementioned range, and the BtP values varied from 47 to 130 kPa, accounting for 176% variation (Fig. 2b). For instance, the measured BtPs at the ramp rates of 0.2 and 65 kPa/s were 47 and 130 kPa and took 235 and 2 s to reach BtP, respectively. Interestingly, the deeper the DRC for a given diameter, the higher the BtP (Supplementary Fig. 4). Notably, the pressure values reported here and elsewhere correspond to the gauge pressure, that is, the absolute pressure minus the atmospheric pressure, unless specifically noted. We explain these trends in the Discussion section.

### Continuous cyclic pressure (no interval)

**Fig. 3:**
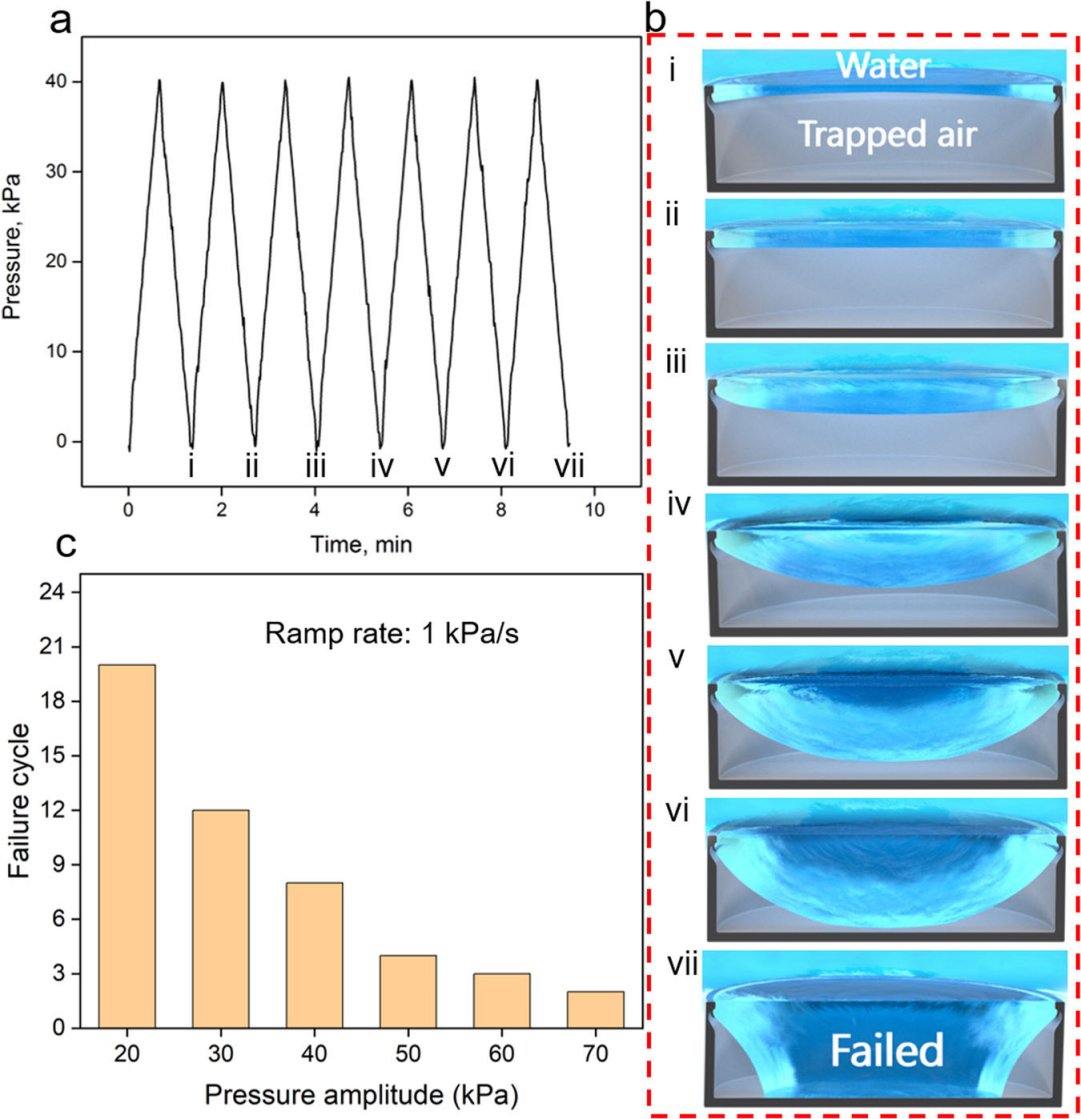
Submerged doubly reentrant cavities under continuous cyclic pressure (*t*_i_ = 0). **a** 40-kPa triangular pressure cycles with zero interval time. **b** Schematic qualitatively illustrates how the interface progresses inside the cavity during each cycle and eventually “fails” (i.e., touches the cavity floor) in the seventh cycle. Scale bar: cavity diameter, *D* = 200 μm. **c** For a fixed ramp rate of 1 kPa/s, as the pressure amplitude increased, the failure cycle number decreased.

In the next experiment, the silica GEMS were submerged in 3-mm-thick water columns, and the headspace was subjected to cyclic pressure in the form of triangular waves separated by a zero interval (*t*_i_ = 0) (Fig. 3a). The pressure amplitude ranged from 20 to 70 kPa, pressurization/depressurization rate was set at 1 kPa/s, and fate of the air trapped inside the DRCs was recorded. Figure 3b presents the representative behavior of the air–water interface under 40-kPa cyclic pressure (Supplementary Video 2). Because the pressure amplitude was lower than the BtP, water remained pinned at the DRC edge; meanwhile, the water–vapor interface reduced inside the cavity but did not touch the cavity floor. In this process, the interfacial curvature of the air–water interface changed from being flat to concave with increasing pressure. Pinning of the interface at the DRC edge was confirmed via confocal laser scanning microscopy (Supplementary Figs. 5, 6 and Supplementary Note 1). However, on full depressurization, the interface did not recover to its original position and following each cycle, it continued to droop into the DRC and eventually touched the cavity floor (after six cycles). Here, we defined the failure cycle as the number of pressure cycles that was needed for the interface to touch the cavity floor (Fig. 3b(v)). With increasing pressure amplitude (ramp rate: 1 kPa/s), DRCs failed rapidly, that is, they touched the cavity floor in less number of cycle (Fig. 3c). We explain these trends in the Discussion section.

### Cyclic pressure with intervals

**Fig. 4:**
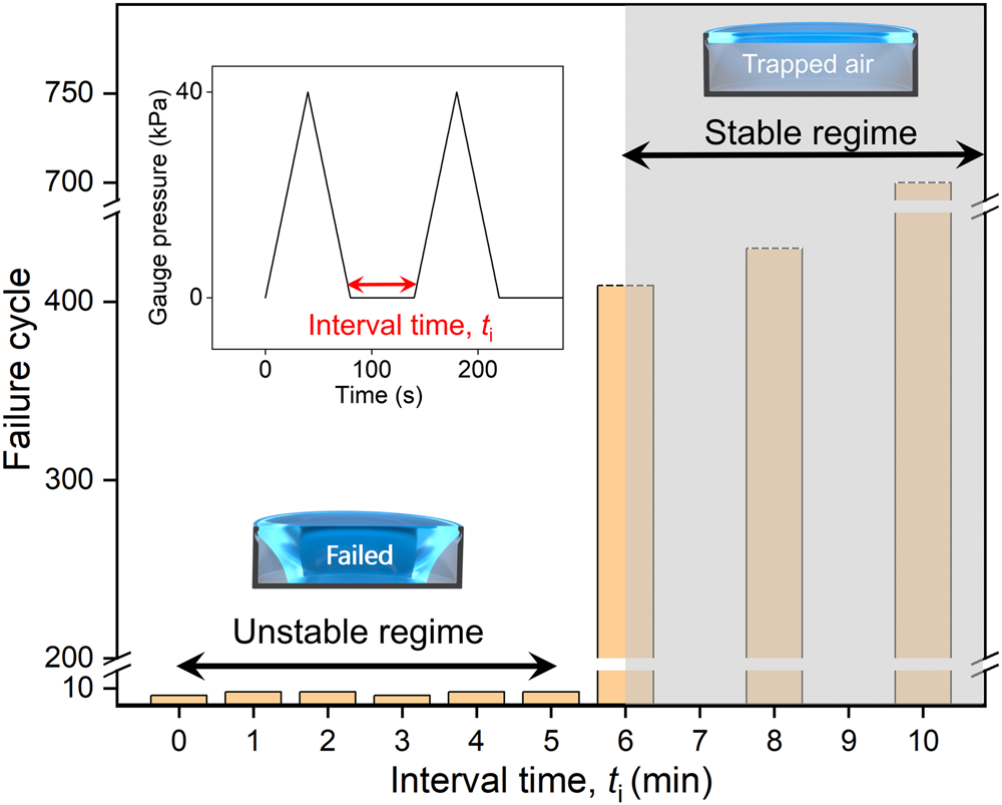
Submerged doubly reentrant cavities (DRCs) under cyclic pressure with intervals (*t*_i_ > 0). Dramatic effects of time interval *t*_i_ during 40-kPa triangular pressure cycling on the fate of the air trapped inside the DRC submerged under a 3-mm-thick water column. For *t*_i_ ≤ 5 min, DRCs failed within eight cycles. In contrast, for *t*_i_ ≥ 6 min, DRCs became immune to air depletion; the air was intact even after >400 cycles, after which the experiments were discontinued.

Further, we modified the above-described experiment by simply separating the triangular pressure waves of 40 kPa amplitude in time intervals *t*_i_ of 0, 1, 2, 3, 4, 5, 6, 8, or 10 min (Fig. 4 inset). The effect was profound; depending on *t*_i_, cavity dimensions, and water column height, two extreme regimes emerged: (i) the unstable regime, wherein *t*_i_ did not considerably influence the failure cycle number for DRC and it failed within a few cycles (data for *t*_i_ ≤ min in Fig. 4 and Supplementary Video 3), and (ii) the stable regime, where *t*_i_ delayed the cavity failure indefinitely (data for *t*_i_ ≥ 6 min in Fig. 4 and Supplementary Video 3). Specifically, for *t*_i_ ≥ 6 min, the air trapped inside the DRCs became immune to depletion as it is pinned at the DRC edge and air–water interface flopped up and down with the cyclic pressure for over 400 cycles (48 h) and did not deplete at all; subsequently, experiments were discontinued. In addition, after pressure cycling in the stable regime, BtP was found to be remarkably higher; for instance, after 700 cycles of triangular waves with 40-kPa pressure amplitude and *t*_i_ = 10 min, BtP increased by 28% (Supplementary Fig. 7). To the best of our knowledge, such DRC behavior has never been reported before, and we will explain the underlying mechanisms in the Discussion section.

### Positive, negative, and alternating positive–negative pressure cycles

**Fig. 5:**
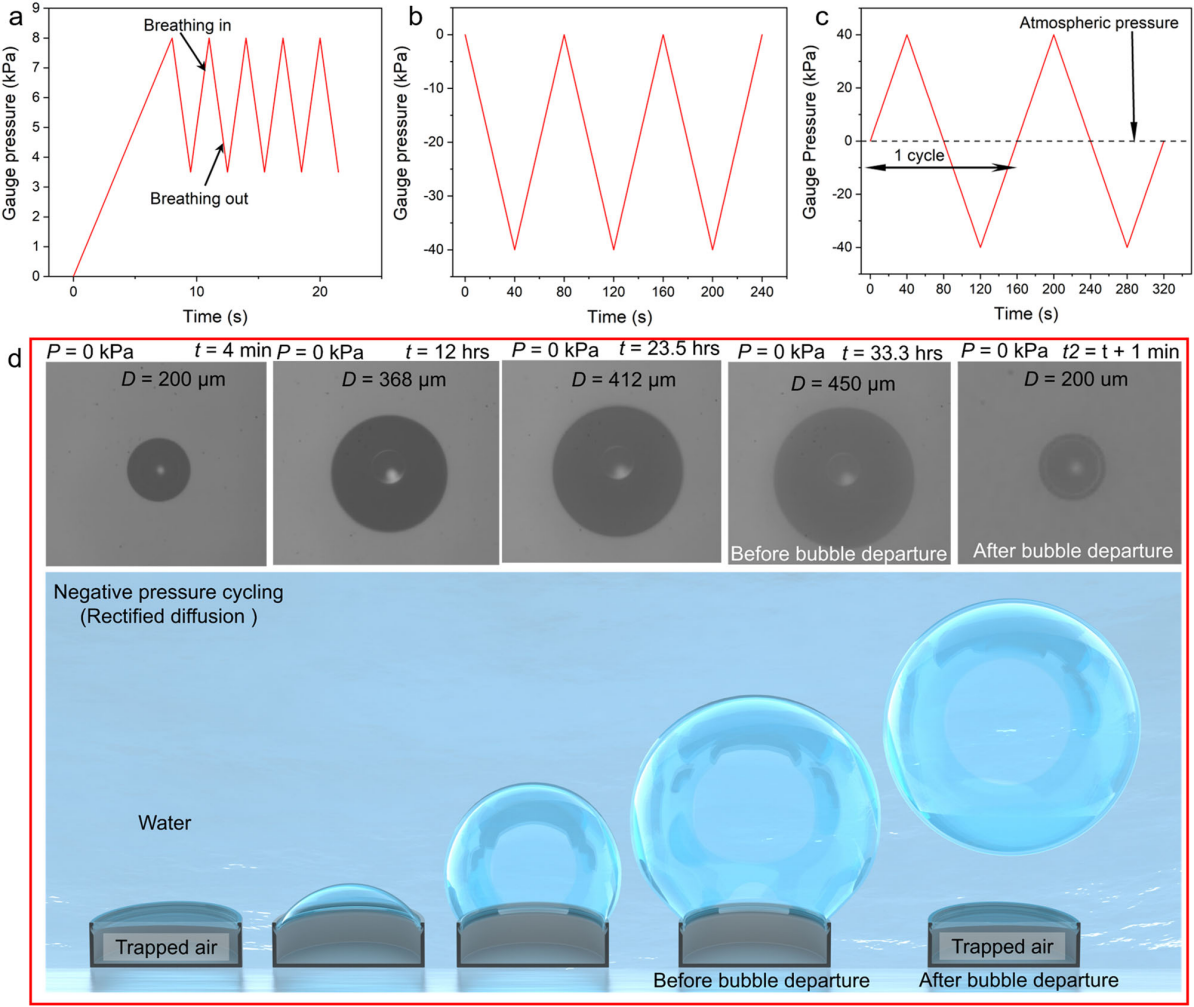
Submerged doubly reentrant cavities (DRCs) subjected to positive (>1 atm), negative (<1 atm), and alternating positive–negative (≷ atm) pressure cycles. **a** Positive pressure cycles akin to basal human breathing. **b** Negative pressure cycles. **c** Positive and negative pressure cycling. **d** Bubble diameter increased to 450 µm under continuous negative cycles for 33 h, although it remained pinned at the DRC mouth, and DRC exhibited a stable interface even after the bubble rupture due to mechanical vibration. Top panel shows the optical images of the bubble growth sequence (top view), and the bottom panel shows their schematics. Water column height: 3 mm. Scale bar: cavity diameter, *D* = 200 μm.

We broadened our investigation to three pressure cycling scenarios relevant to natural and applied contexts (Fig. 5a–c). They were (i) positive pressure cycling (Fig. 5a), similar to human breathing with continuous positive airway pressure (CPAP)^61^; (ii) negative pressure cycling (Fig. 5b), i.e., reducing pressure below the ambient pressure; and (iii) alternating positive–negative cycles (Fig. 5c), similar to bubbles in acoustic fields and fluctuating flows over surfaces^51,53,56,62^. For the CPAP-type cycling, the breathe-in pressure was set to 8 kPa, breathe-out pressure was set to 3.5 kPa, breathing duration (in/out) was set to 1.5 s, and time interval between each cycle was 0 s (Fig. 5a and Supplementary Video 4). The following two water column heights were tested, *H* = 2 and 6 mm. For the 6-mm water column, DRC failed after 36 min (720 cycles). Remarkably, when the water column height was reduced to *H* = 2 mm while keeping the other experimental parameters the same, cavity failure was prevented for over 40 h (48,000 cycles), after which the experiment was discontinued. We explain the physics behind this 6600% enhancement in the fail time in the Discussion section below. In addition, we conducted a BtP test after 40 h of cycling and found it to be 87 kPa, which was 16% higher than that immediately after immersing DRC in water.

For the second scenario, the GEMS were immersed in 3-mm-thick water columns and subjected to negative triangular pressure cycling amplitude of −40 kPa via a vacuum suction device (the ramp rate was ± 1 kPa/s and *t*_i_ = 0) (Fig. 5b). During each cycle, the headspace pressure (Fig. 2a) was reduced from 101 to 61 kPa. For the trapped air inside the DRC, this pressure change was equivalent to falling from *P* = 0 to *P* = − 40 kPa (Supplementary Fig. 8). Under these conditions, the trapped bubble expanded and shrank; with an increasing number of cycles, it grew out of the cavity (Fig. 5b, d and Supplementary Video 5). At *t* = 33.3 h and *P* = 0 kPa, the diameter of the bubble became 450 µm, even though it remained pinned to the DRC edge. We induced mechanical vibrations to check the mechanical stability of the interface, that is, whether DRC would be catastrophically filled if a significant portion of the bubble departed. Remarkably, as a major portion of the bubble departed, GEMS recovered their initial Cassie state (Fig. 5d and Supplementary Video 6). At this point, the measured BtP was ~75 kPa for a ramp rate of 1 kPa/s.

Finally, the headspace above the GEMS immersed in 3-mm-thick water columns was subjected to alternating positive–negative pressure cycles of amplitude ± 40 kPa (at a ramp rate of ± 1 kPa/s and *t*_i_ = 0) via a combination of positive air pressure and vacuum suction (Figs. 2a, 5c). Intuitively, one would expect that if the same pressure amplitude is applied above and below the baseline, the overall effect on the bubble size should be null. Counterintuitively, the bubble grew in size over time (Supplementary Video 7). We induced mechanical vibrations to detach the enlarged bubble from the cavity. After its departure, GEMS recovered their initial Cassie state.

## Discussion

**Fig. 6:**
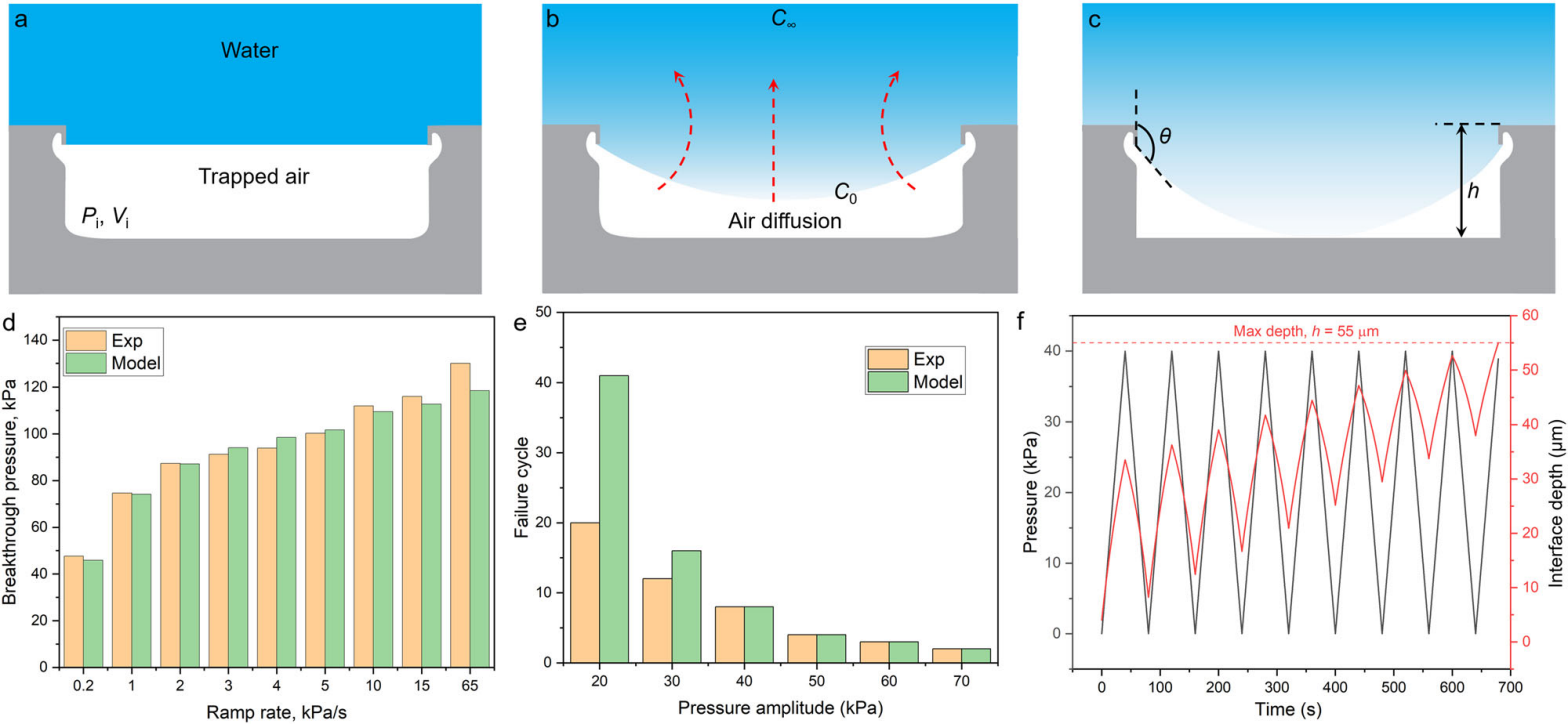
Predicted BtPs and failure cycles. **a**–**c** Schematics of the air–water interface at/inside the doubly reentrant cavity **(**DRC) with respect to increasing pressure. **d** Comparison of predicted BtPs with experimentally observed values for submerged DRCs as a function of the pressure ramp rate. **e** Comparison of predicted failure cycles with experimentally observed values for submerged DRCs as a function of pressure amplitude. **f** Predicted distance between the DR edge and liquid–vapor interface depth (referred to as the interface depth) for a 40-kPa cyclic pressure.

Here, we draw together all the results and discuss the factors and mechanisms underlying the stability of the gas entrapped in silica GEMS under fluctuating pressures. First, we discuss the effects of the pressure ramp rates on BtP (Fig. 2b). As the headspace is pressurized, water tends to enter the cavity and compress the trapped air. Over time, this air dissolves into water column and is transported via diffusion, creating space for water to enter/occupy (Fig. 6a–c). Therefore, a slow ramp rate facilitates easy liquid penetration and yields a low BtP. In contrast, if the ramp rate is high, the dissolution of the trapped air into the water column will be less and liquid penetration will be resisted by gas compression, increasing BtP. We used the ideal gas equation^63^ to predict the effect of the applied gauge pressure *P* on the gas volume inside the cavity *V*, assuming isothermal conditions:

1 $$V={V}_{{{{{{\rm{i}}}}}}}\frac{{P}_{{{{{{\rm{i}}}}}}}}{{P}_{{{{{{\rm{i}}}}}}}+P},$$

where ${V}_{{{{{{\rm{i}}}}}}}$ and ${P}_{{{{{{\rm{i}}}}}}}$ are the initial gas volume and external pressure, respectively. ${P}_{{{{{{\rm{i}}}}}}}$ is defined as ${P}_{{{{{{\rm{i}}}}}}}=({P}_{{{{{{\rm{atm}}}}}}}+\rho {gH}-{P}_{{{{{{\rm{v}}}}}}})$, accounting for the atmospheric ${P}_{{{{{{\rm{atm}}}}}}}$, hydrostatic $\rho {gH}$, and vapor pressures ${P}_{{{{{{\rm{v}}}}}}}$.

To account for the diffusional gas loss from the trapped air, we employed Fick’s first law along with Henry’s law. According to Henry’s law, gas solubility in water increases with increasing gas pressure. Considering air to be a 79:21 mixture of N_2_ and O_2_ gasses, whose Henry’s constants are known, the concentration of dissolved air in the water (${c}_{\infty }$) in equilibrium with the atmosphere at 1-atm pressure is expected to be ${c}_{\infty }$ = 23 g/m^3^.^64,65^. Further, as the water is pressurized, the new equilibrium gas concentration at the air–water interface will be higher than the previous one, which can be calculated using Henry’s law as ${c}_{0} > \,{c}_{\infty }$. This leads to the buildup of a vertical concentration gradient between the interface and bulk, ${c}_{0}-{c}_{\infty }$ and drives gas dissolution as per Fick’s first law, $J=-{D}_{{{{{{\rm{G}}}}}}}\frac{\Delta c}{l}$. Here, *J* is the mass flux, *D*_*G*_ is the diffusion constant, and *l* is the diffusion length. We assumed that the trapped air exhibits ideal gas behavior (${p}_{G}V={nRT}$) and mass flux of the air from the cavity to the water per unit area is $[J=\left(\frac{{{{{{{\rm{d}}}}}}n}}{{{{{{{\rm{d}}}}}}t}}\right)/A(t)]$, where$\,{p}_{G}={P}_{{{{{{\rm{i}}}}}}}+P$ and *V* are the pressure and volume of the trapped air, respectively, $n$ is the number of gas moles in the trapped air, *R* is the universal gas constant, *T* is the temperature, and *A* is the gas–liquid interface area. Based on the above equations, we can rewrite Fick’s first law as (Supplementary Note 2)^66^

2 $$\frac{{{{{{\rm{d}}}}}}\left({p}_{{{{{{\rm{G}}}}}}}(t)V(t)\right)}{{{{{{\rm{d}}}}}}t}=-{RTA}\left(t\right)\frac{{D}_{{{{{{\rm{G}}}}}}}}{{K}_{{{{{{\rm{H}}}}}}}}\frac{\left({p}_{{{{{{\rm{G}}}}}}}\left(t\right)-s{.p}_{{{{{{\rm{G}}}}}},0}\right)}{l},$$

where ${K}_{{{{{{\rm{H}}}}}}}$, $l$, and $s$ are Henry’s constant, diffusion length, and gas solubility, respectively. The pressure inside the cavity is denoted by ${p}_{{{{{{\rm{G}}}}}}}$, while ${p}_{{{{{{\rm{G}}}}}},0}$ is the gas partial pressure at atmospheric pressure.

Equation (1) describes the effect of pressure change on trapped gas volume, while Eq. (2) describes gas volume loss due to diffusion. By numerically solving Eqs. (1) and (2) iteratively for an increasing *P*, we obtained BtP for various pressure ramp rates and failing cycles for various pressure amplitudes. Figure 6d, e compare the experimental results with the theoretical results, demonstrating that the model can successfully capture the physics underlying the experimental observations. The details of conducting the simulation are explained in the Supplementary Information (Supplementary Note 2). Notably, there are some differences between the model predictions and the experimental observations at low pressures (Fig. 6e). This is expected since we only consider the diffusion in one dimension in our model, whereas in reality this happens in 3D.

Increasing the ramp rate gave minimal time for gas diffusion and hence higher BtP. Here, the BtP of common rough surfaces/membranes typically depended on the Laplace pressure, ${P}_{{{{{{\rm{L}}}}}}}$, arising due to the curvature of the air–water interface at the microtexture’s inlet. We estimated the maximum possible contribution of ${P}_{{{{{{\rm{L}}}}}}}$ for our DRC using the expression ${P}_{{{{{{\rm{L}}}}}}}=-4\gamma \cos {{{{{{\rm{\theta }}}}}}}_{\max }/D$. Interestingly, ${P}_{{{{{{\rm{L}}}}}}} \sim 1\,{{{{{\rm{kPa}}}}}}$ was insignificant compared with the air pressure inside the DRC generated due to its compression, which underscores the importance of the entrapped gas and its time-dependent loss on BtP.

The effects of pressure ramp rates on BtPs are relevant to underwater breathing insects, such as the alkali fly (*Ephydra hians*), which can crawl to 4–8-m-deep waters for foraging and laying eggs^12^. It could maximize plastron respiration by rapidly diving deep to minimize the loss of trapped gas or simply staying in shallow water where the hydrostatic pressure is low and wetting transitions are slow. A case-by-case assessment of various animals is warranted for biomimicry.

Next, we explain the emergence of unstable and indefinitely stable regimes under pressure cycling and how they depend on *t*_i_, water column height, and cavity dimensions (Figs. 3 and 4). Here, we first need to decouple the contributions of the dissolution and diffusion of the trapped air and the headspace air into the water column during pressurization and depressurization. During continuous pressure cycling or cycles separated by *t*_i_ ≤ 5 min for the DRCs studied herein, trapped air is lost via diffusion into water due to pressure-induced undersaturation. Hence, the liquid meniscus approaches the cavity floor monotonically and air entrapment falls in the unstable regime. The gradient of dissolved gas concentration in undersaturated water $({c}_{o}-{c}_{\infty })$ > 0 results in a continuous outward mass flux (*J* < 0) until trapped air dissolves in water. Using Eqs. (1) and (2) but for the cyclic variation of *P*, we captured the failure cycle due to cyclic pressure with different pressure amplitudes (Fig. 6e). Figure 6f shows the failure cycle for a 40-kPa-amplitude cycle and the corresponding distance between the DR edge and the liquid–vapor interface depth, which we refer to as the interface depth. This reveals that the interface depth increases for each cycle, indicating that the gas volume is lost through diffusion at each cycle.

**Fig. 7:**
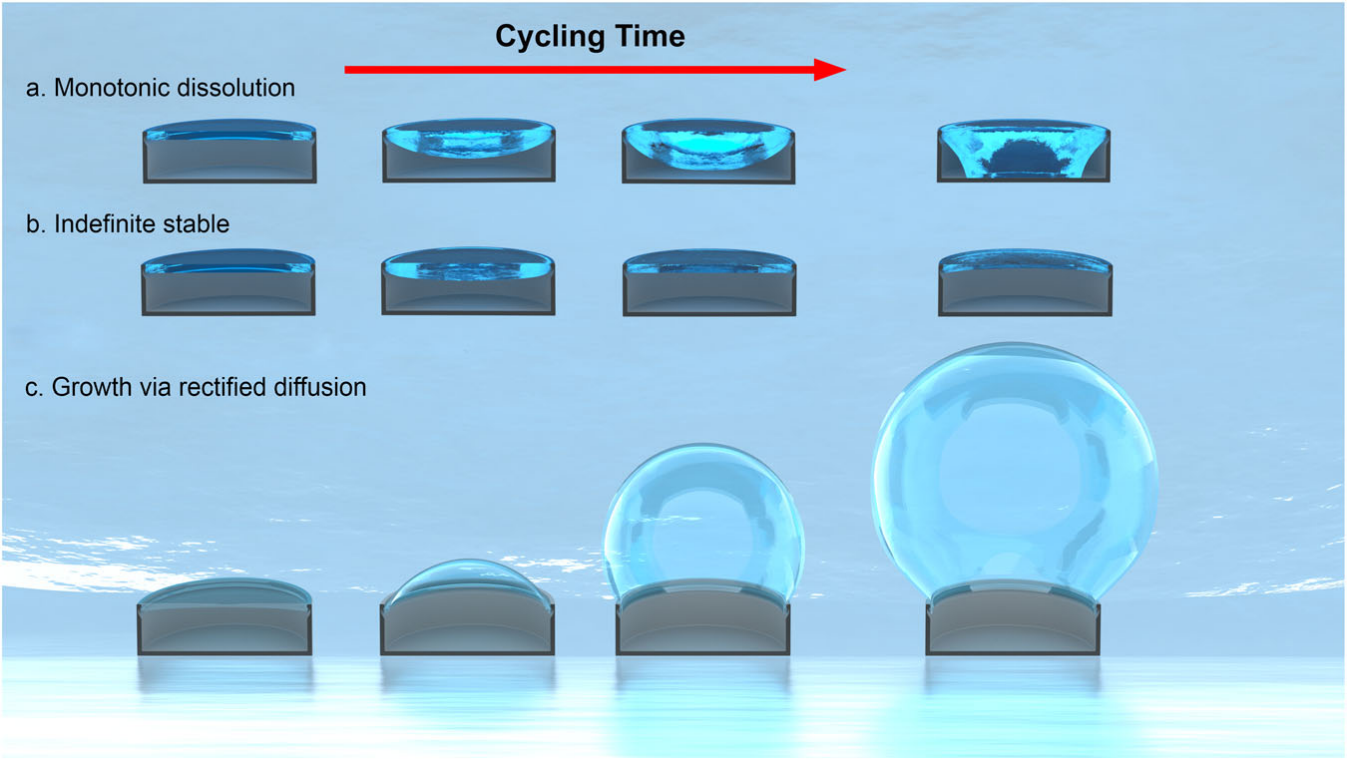
Summary of trapped bubble stability under cyclic pressure. **a** Trapped air monotonically depletes because of diffusion under continuous positive cycles in undersaturated water. **b** Trapped air is indefinitely stable under positive cycling in saturated water. **c** Trapped bubble starts growing because of rectified diffusion under continuous negative and positive–negative cycles. Scale bar: cavity diameter, *D* = 200 μm.

By contrast, the indefinitely stable regime for the trapped air in the DRCs studied in this work is observed when the cycles are separated by *t*_i_ ≥ 6 min (Fig. 4). This interval time enables water column to become saturated under the applied pressure via air diffusion from the headspace. This results in $({c}_{o}-{c}_{\infty })=0$, leading to no mass flux (*J* = 0) and no cavity failure. In our experiments, as well as for insects under shallow waters^2^, gas exchange at the headspace controls the water saturation level, dictating whether bubble would “breathe” outward or inward. The time dependence of the spatial gas concentration is governed by Fick’s second law, $\frac{\partial c}{\partial t}={D}_{{{{{{\rm{G}}}}}}}\frac{{\partial }^{2}c}{\partial {z}^{2}}$^64,66,67^. Considering I–D diffusion for simplicity, the time required to saturate the entire water column height can be estimated using the equation *t*_D_ ≈ $\frac{{z}^{2}}{2D}$ for the 3-mm water column and it may take *t*_D_ ≈ 38 min to saturate water under hydrostatic conditions^64^. This means that for the air trapped inside the DRC under a specific liquid column height *H* and subjected to pressure cycling, if the interval time *t*_i_ is such that the time for cavity failure cycle is greater than *t*_D_, then the water column becomes saturated before water touches the DRC floor. In such a scenario, the curvature of the saturated air–water interface diffuses air into the cavity until its curvature is lost (i.e., it flattens). Because our experiments entailed pressure cycling, the actual time to saturate the water column was longer (positive pressure cycles; Fig. 4), i.e., <60 min. To test this, we varied the water column height from 3 to 6 mm and subjected it to a 40-kPa cyclic pressure (with a ramp rate of 1 kPa/s and *t*_i_ = 6 min). For the 3-mm column height, the system acquired a stable state; meanwhile, for the 6-mm column, DRC failed at the seventh cycle. If we reduced the water column height to 2 mm, the stable regime could be reached under *t*_i_ = 5 min under a 40-kPa cyclic pressure. This underscores the importance of the DRC (or bubble) size, *t*_i_, and *t*_D_ in achieving water column saturation and realizing breathing interfaces. This also provides clues for bubble stability in aquatic beetle (*P. tuberosus*), which lives in shallow flowing waters and is known to be saturated with air^2^. Figure 7a, b summarizes the behaviors of trapped bubbles under positive cyclic pressures with zero and nonzero *t*_i_. According to Henry’s law, pressurizing water above atmospheric pressure makes it undersaturated $\left({c}_{o}-{c}_{\infty }\right) > 0$, with respect to the gas bubble; while, depressurizing it below atmospheric pressure results in supersaturation $\left({c}_{o}-{c}_{\infty }\right) < 0$ with respect to the bubble (Fig. 7c; discussed below). For positive cyclic pressures, careful selection of *t*_i_ and *t*_D_ may result in the stable regime, wherein bubble may exhibit indefinite stability. In this study, we used a vertical overhang length (*h*_DL_) of 4 µm, accounting for approximately 7% of the cavity depth (*h* = 55 µm). Our model predicts that for a constant cavity depth, increasing *h*_DL_ decreases BtP and the failure cycle number (Supplementary Fig. 9). In addition, in this study, we used shallower cavities (*h*/*D* < 1) to ensure that the liquid–vapor interface remained pinned to the DR edge. As the pressure increased, the curvature of the spheroid-shaped water–vapor interface continued to increase until the water touched the cavity floor. However, a depinning mode of failure^44,68^ could be expected for deeper cavities (*h*/*D* > 1) wherein an increase in the hydrostatic pressure causes the primary meniscus to reach its maximum curvature (≈*D*/2) and depins from the DR edge onto the cavity walls. As a result, the advantages of the DR features were lost, and DRCs behaved as simple cavities^50^.

Next, we explain the curious observation of bubble growth under continuous negative and positive–negative pressure cycling, i.e., *t*_i_ = 0 (Fig. 5b, c). While bubble growth is intuitive for purely negative cycles, it is not so obvious for positive–negative pressure cycling as one would expect the bubble size to remain unchanged. In a typical cycle, as the pressure in the headspace drops, the water column becomes oversaturated, which transfers air into the entrapped gas pocket (and into the headspace). In oversaturated water, $\left({c}_{o}-{c}_{\infty }\right) < 0$ results in an inward mass flux (*J* > 0). Interestingly, research has revealed that when a bubble is exposed to pressure cycling within a supersaturated liquid, it may start growing if the pressure amplitude is beyond a certain threshold, a phenomenon known as rectified diffusion^69,70^. Although a detailed investigation of this process is beyond the scope of this study, scientific literature suggests that rectified diffusion occurs due to the nonlinear combination of the enhanced surface area during expansion (area effect) and the “shell effect,” which refers to the difference in the thicknesses of the liquid–gas mass transfer boundary during expansion and compression^71^. During compression, this boundary layer is thicker than that during expansion, promoting the diffusion of more gas in the bubble during expansion than during compression. For an insect respiring via plastron under water, it is thus advantageous to be in shallow flowing water because (i) it is saturated with oxygen and, (ii) during exhalation, it can register a net gain of O_2_ gas^2^ as the plastron expands. More work is needed to determine whether the pressure amplitudes (positive/negative/positive–negative) and the frequency of breathing are adequate for rectified diffusion^71^.

Finally, we explain the significantly higher BtPs recorded after the pressure cycling experiments. This is related to the gas oversaturation of water during positive pressure cycling such that the system enters the stable regime (Fig. 4). If a BtP measurement follows, as water is pushed into the cavity, the gas from the cavity dissolves much more slowly into the water due to its oversaturation, which manifests as a higher BtP (Supplementary Fig. 7).

## Conclusion

To the best of our knowledge, this is the first report on the effects of pressure cycling on DRC-based GEMS, and the findings are certainly relevant to hydrophobic and superhydrophobic surfaces/membranes that serve many practical applications. Before this report, DRCs immersed in water were known to monotonically lose entrapped air due to the coupling between capillary condensation and gas diffusion^39,72^. The current results unveil a range of hitherto unrealized possibilities for the air trapped inside submerged DRCs under pressure cycling, such as enhanced BtP, unstable states, indefinitely long stable Cassie states, bubble growth due to rectified diffusion, and recovery of the original Cassie state after losing the bubble, depending on the pressurization/depressurization rates, *t*_i_, *t*_D_, water column height, and DRC volume. These results may also provide clues to understanding air-breathers’ underwater microtexture, depths and durations explored, diving speeds, plastron stability under continuous and discontinuous breathing cycles^73,74^, and rectified diffusion. Our experimental model system may also facilitate the simulation of pressure cycling for human divers’ safety, which is otherwise studied via acoustic bubble growth at low frequencies^75^. Under the constraints of present and future regulations on perfluoroalkyl substances^46,47^, DRCs may afford a coating-free alternative for applications that require omniphobicity. Proof-of-concept demonstrations for DRCs been recently reported under hydrostatic scenarios, which have helped in mitigating cavitation erosion^19^, enhancing heat transfer^34,76^, microfluidics applications^77^, separation and purification technologies^48,78^, frictional drag reduction^79^, and oscillating-bubble-powered microswimmer applications^80^. As the prospect of translating these interesting findings into practical solutions matures, concerted efforts will be needed to investigate the effects of pressure cycling on GEMS of varying depth, as well as the collective behavior of DRC arrays, with a wide range of probe liquids, especially those with low surface tensions.

## Methods

### Microfabrication

We used a layout designer (Tanner L- edit, V15.0) to create the cavity pattern, and a direct writer (Heidelberg Instruments, µPG501) exposed the design to a 1.6-µm-thick AZ5214E spin-coated SiO_2_/Si wafer (Silicon Valley Microelectronics, orientation 100, single-side polished, 4-in. diameter, 500-µm thickness, 2-µm thick oxide layer). The UV-exposed photoresist was removed in an AZ-726 developer bath. The SiO_2_ and Si layers were etched using inductively coupled plasma (ICP) reactive-ion etching (RIE) and deep ICP–RIE (Oxford Instruments), respectively. In between these etching steps, a 500-nm-thick thermal oxide was grown over the etched wafer using a Tystar furnace system to realize a doubly reentrant edge. After etching to the required depth, the samples were cleaned using a piranha solution (H_2_SO_4_/H_2_O_2_ = 3:1 by volume) at *T* = 115 °C for 10 min and spin-dried in a nitrogen environment. The wafer was then stored in a glass Petri dish and placed inside a clean vacuum oven maintained at *T* = 50 °C and gauge pressure *P*_vac_ = −80 kPa for 5 days, after which the intrinsic contact angle of the silica layer stabilized to *θ*_o_ ≈ 63°. We adopted this microfabrication procedure from our previous work^37,39,45^.

### Scanning electron microscopy (SEM)

We used Quattro (from Thermo Fisher Scientific) for SEM imaging. Microtextured samples were mounted on a stub using double-sided carbon tape. Furthermore, samples were coated with 3 nm of iridium using a sputter coater (Quorum Q150T S) before SEM analysis to eliminate charging effects.

### Contact angle measurement

The apparent water contact angles of samples were measured using a Kruss Drop Shape Analyzer (DSA100E). Liquid advanced/receded at a rate of 0.2 μL s^−1^. All data were analyzed using the *Advance* software, and the reported data points are the average of at least three measurements.

### BtP and cyclic pressure experiment

We fixed the sample into a Petri dish (35-mm petri dish from Celltreat) using permanent double-sided tape (scotch) and kept it inside a custom-built pressure cell. Before the experiment, we added water (≈3 mL at a rate of ≈0.3 mL/s) using a syringe and needle to submerge the sample and closed the pressure cell with a top cover. We used a Fluigent pressure controller (push and push & pull type), Keller (LEO3 digital manometer) pressure gauge, and compressed air/vacuum to increase or decrease the headspace pressure. The required pressure ramp rate and cyclic pressure profile were programmed using microfluidic automation software provided by Fluigent. The default pressure ramp rate was set to 1 kPa/s unless specified otherwise. Wetting transitions inside the cavity were observed from the top using a monochrome digital camera (Edmund Optics, EO-5310) attached to a Qioptiq objective that has a working distance of 9.5 cm. We used a screen recorder (VSDC) to continuously capture the ensuing wetting transitions and later analyzed them to extract BtP, failure time, and failure cycle details. Unless stated otherwise, all pressure values reported here correspond to gauge pressure.

### Numerical calculations

The numerical simulations were performed using the Mathematica software. The details of the algorithm and parameter values are provided in Supplementary Note 2.

### Supplementary information

Supplementary Information

Description of Additional Supplementary Files

Supplementary Video 1

Supplementary Video 2

Supplementary Video 3

Supplementary Video 4

Supplementary Video 5

Supplementary Video 6

Supplementary Video 7

## Data Availability

All data related to the study are included in the article and/or Supplementary information.

## Code availability

Mathematica code used in the current study is available from the corresponding author upon reasonable request.
