# Supplementary material for: Insect-inspired breathing interfaces: investigating robustness of coating-free gas entrapping microtextured surfaces under pressure cycles
Source: Commun Eng. 2024 Jun 21;3:84. doi: 10.1038/s44172-024-00231-2 (PMC11192747; doi:10.1038/s44172-024-00231-2)
Supplement: Supplementary file 1 — Supplementary Information [file 44172_2024_231_MOESM1_ESM.pdf]

# Supporting Information

**Insect-Inspired Breathing Interfaces: Investigating Robustness of Coating-Free Gas Entrapping Microtextured Surfaces Under Pressure Cycles**

Sankara Arunachalam[1,2*], Muhammad Subkhi Sadullah[1,2] & Himanshu Mishra[1,2,3*]

[1]Environmental Science and Engineering Program, Biological and Environmental Sciences and Engineering Division, King Abdullah University of Science and Technology (KAUST), Thuwal 23955-6900, Kingdom of Saudi Arabia

[2]Water Desalination and Reuse Center, King Abdullah University of Science and Technology, Thuwal, Kingdom of Saudi Arabia

[3]Center for Desert Agriculture, King Abdullah University of Science and Technology, Thuwal, Kingdom of Saudi Arabia

[*]Corresponding authors: sankara.arunachalam@kaust.edu.sa & himanshu.mishra@kaust.edu.sa

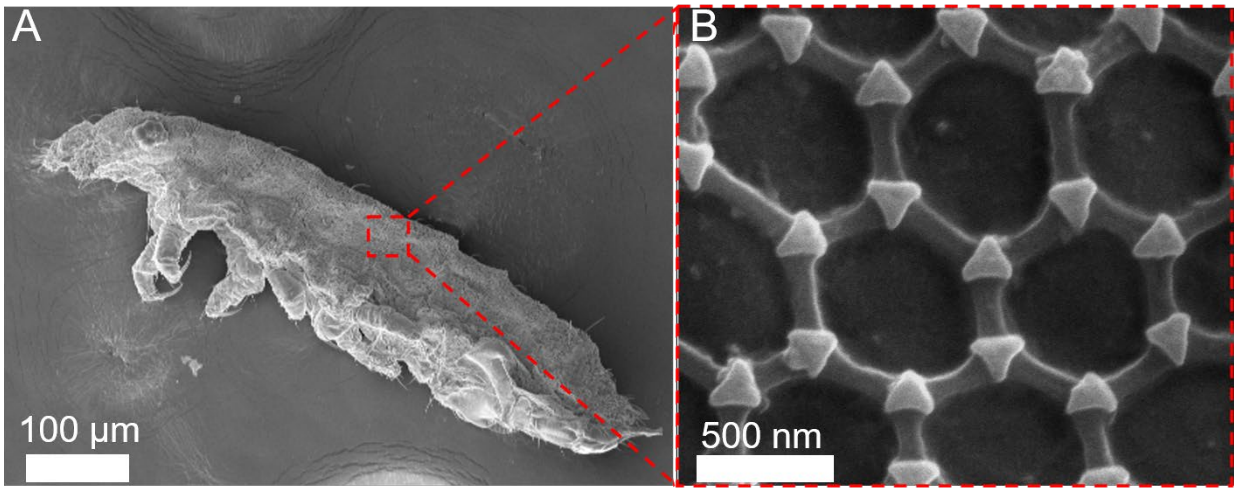


**Supplementary Figure 1:** Representative scanning electron micrographs of the cuticles of springtails (Collembola) covered by a layer of nanoscopic interconnected granules forming a basic hexagonal pattern.

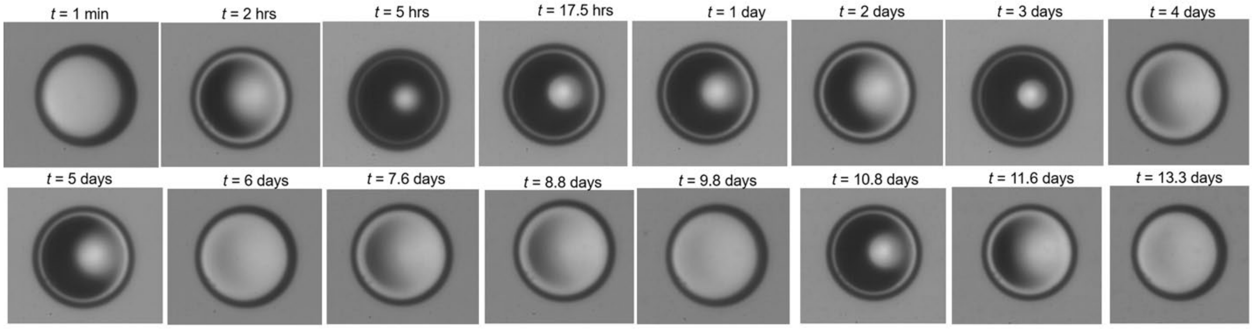


**Supplementary Figure 2:** Optical micrographs (top view) of silica surfaces with a circular doubly reentrant cavity immersed in a 5-mm water column. Intrinsic contact angle of flat silica in air is $\theta_o \approx 63^{\circ}$. No failure is observed until 13 days. Cavity diameter is 200 μm and depth is 67 μm.

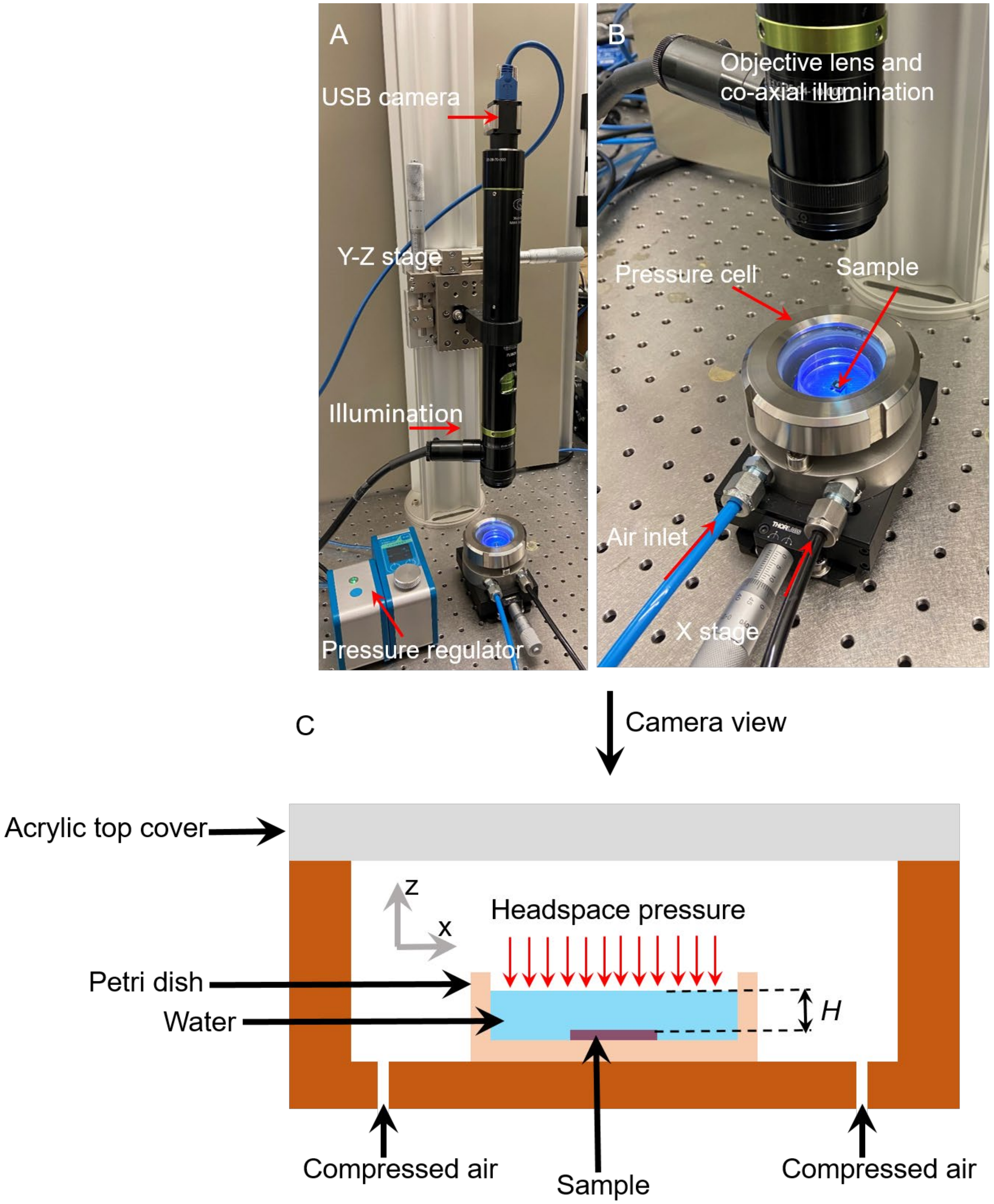


**Supplementary Figure 3:** (A) Experimental setup to determine the breakthrough pressure (BtP) of cavities with water. Optical images were recorded using a vertically mounted microscope attached to a USB camera. (B) Samples were immersed in water inside a pressure cell made of stainless steel and with a polymethylmethacrylate top cover. Subsequently, we used compressed air to apply controlled external pressure via a fluigent pressure regulator. (C) Schematic of the pressure cell, where $H$ is the water column height above the sample.

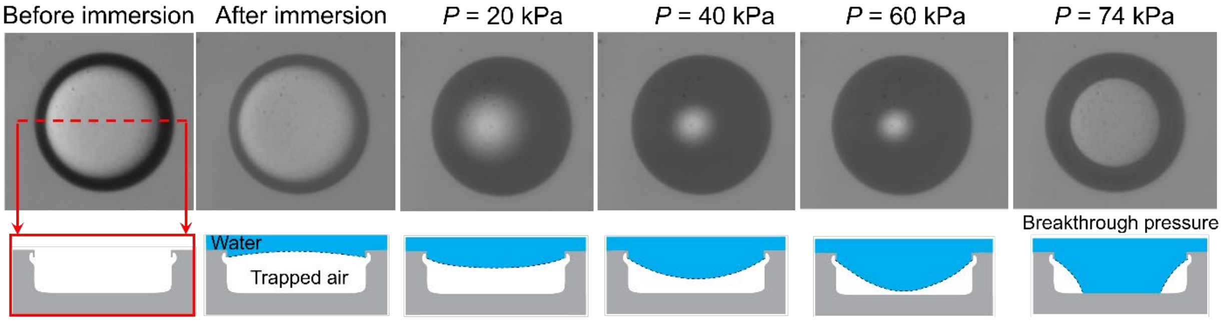


**Supplementary Figure 4:** BtP was characterized under 3-mm-thick water columns by gradually ramping up the pressure at a rate of 1 kPa/s. Top panel shows optical micrographs (top view) of wetting transitions in a single doubly reentrant cavity under increasing hydrostatic pressure. Bottom panel shows the gas–liquid interface inside the cavity under increasing pressure (scale bar: cavity diameter $D$ = 200 μm). For cavity depth $h$ = 55 μm ($h$ = 67 μm), BtP was found to be 73.5 kPa (79 kPa).

## Supplementary Note 1: Confocal Imaging

To achieve high-resolution visualization of the air–water interface under pressurization and depressurization, we built a custom-built pressure device to use confocal laser scanning microscopy (CLSM). After placing the sample's Petri dish in the pressure cell, a syringe was used to gently pour water (containing 0.01-M rhodamine B dye) until the sample was completely submerged. To minimize localized heating, we kept the laser intensity as low as possible (0.2 mW) and captured sequential line scan images in the Z-stack mode along the cavity diameter. Line scan mode was used instead of two-dimensional scan to acquire images faster with minimal heating.

In a representative positive pressure cycling experiment, we increased the pressure to 20 kPa with a ramp rate of 1 kPa/s, held it for 1 min for imaging, and then depressurized it. This process was repeated after a 3-min interval. After a few cycles, the pressure was increased to 40 kPa (Supplementary Figs 5 and 6). When DRC was immersed in water, i.e., without applied pressure, the intruding air–water interface was initially slightly convex at the DR edge (Fig. S6A). Subsequently, with the application of the external pressure from 0 to 20 kPa, the air–water interface started to become flat and sagging inside (concave) the cavity (Supplementary Fig. 6B). Additionally, as the pressure was relieved back to the ambient condition, the air–water meniscus began to move upward and reached a flat position, confirming a remarkable diffusional loss of

air from the cavity (Supplementary Fig. 6C). As the number of cycles increased, the diffusional air loss from the cavity increased, which is evident from the meniscus shape (Supplementary Fig. 6D–F). With a higher-amplitude pressure cycle (40 kPa), the diffusional loss increased and meniscus touched the cavity floor (failed; Supplementary Figs. 6G–L). Interestingly, throughout these wetting transitions, water meniscus was in contact with the DR edge as it remained pinned to the DRC mouth.

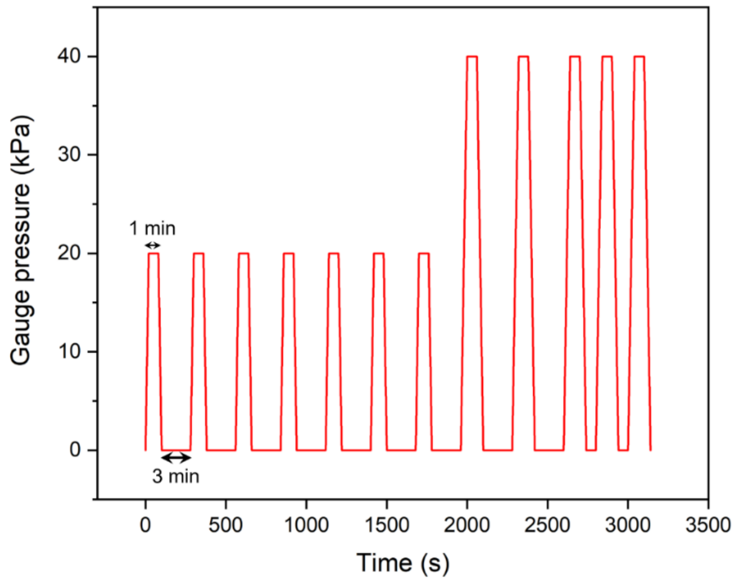


**Supplementary Figure 5:** Cyclic pressure pattern used for obtaining the confocal images presented in supplementary fig. 6A–L.

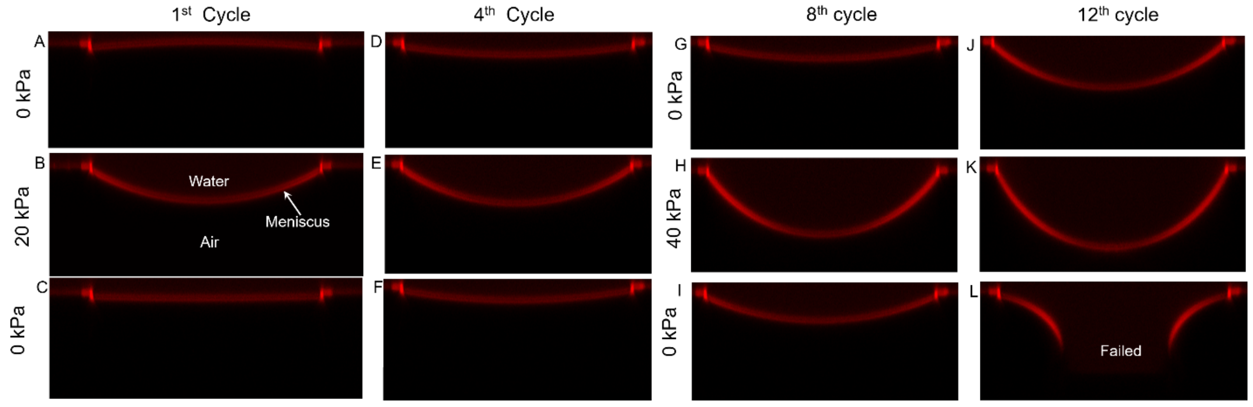


**Supplementary Figure 6:** (A-F) Confocal micrographs of the wetting transitions of silica surfaces with a doubly reentrant cavity under 20-kPa cyclic pressure, (G-L) followed by 40-kPa cyclic pressure, as shown in supplementary fig. 5. We used the line scan mode along with Z-stack to image across the diameter of the cavity (scale bar: cavity diameter $D$ = 200 µm).

Here, we would like to point out that the heat from the CLSM laser can accelerate wetting transitions with water, so only qualitative trends can be drawn[1,2]. In response, we used upright optical microscopy, which does not suffer from these artifacts but lacks resolution and visualization. Thus, CLSM and upright optical microscopy are complementary in investigating wetting transitions.

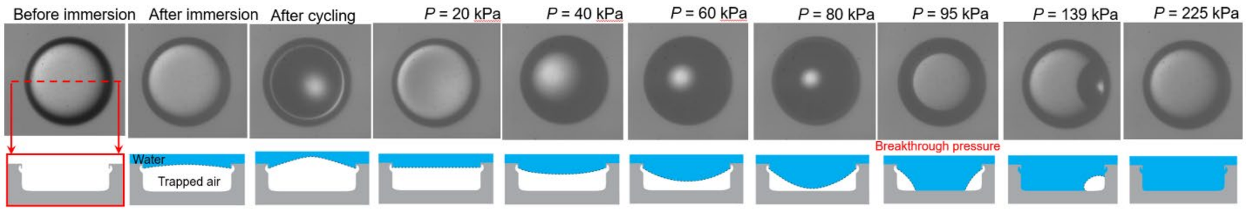


**Supplementary Figure 7:** Enhancement in BtP after the cyclic pressure experiment. After 700 cycles of 40-kPa pressure amplitude with 10-min intercycle time, BtP increased by 28%. Water was oversaturated than the initial conditions (scale bar: cavity diameter $D$ = 200 μm and depth, $h$ = 55 μm).

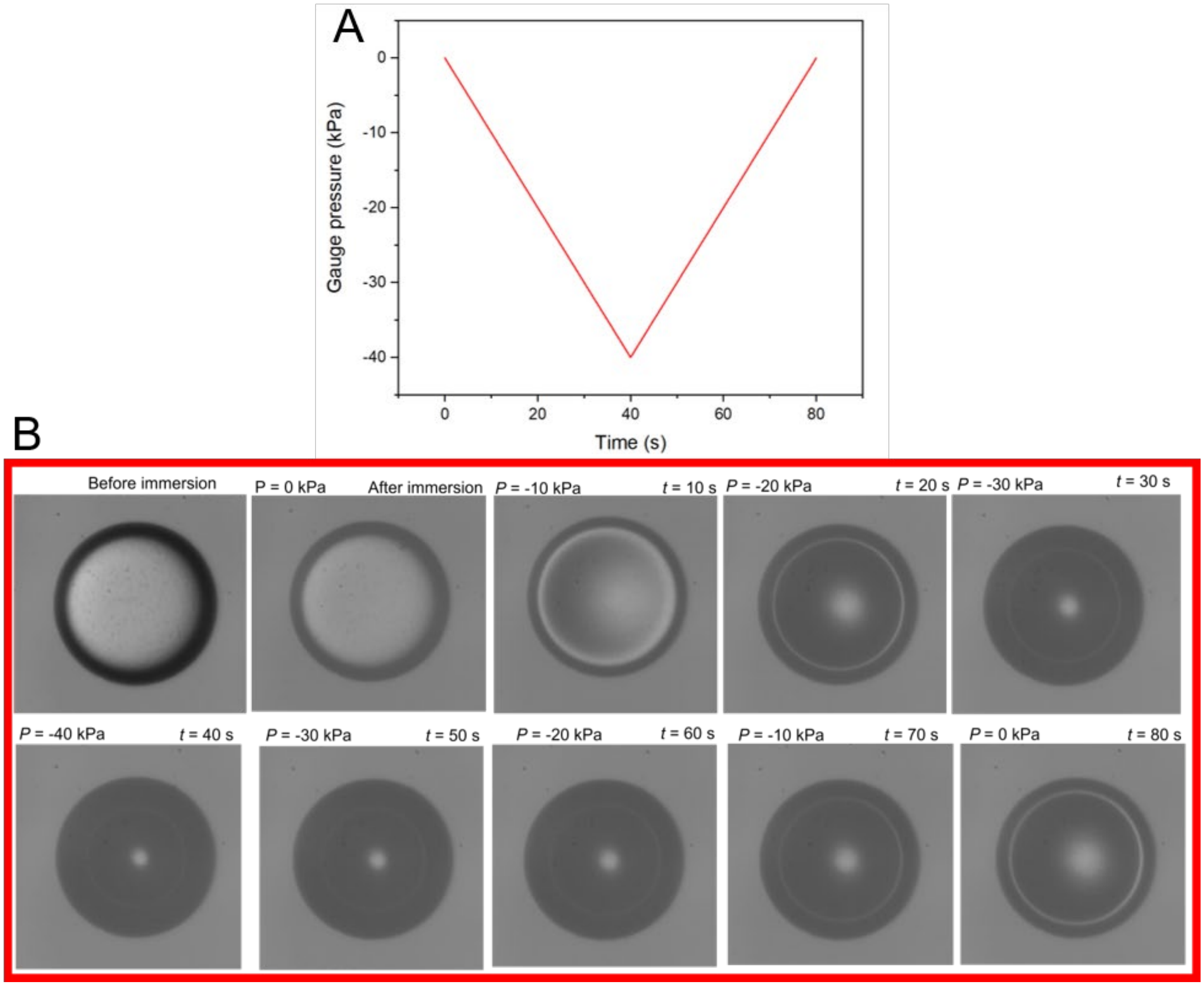


**Supplementary Figure 8:** Top-view optical micrographs of a single 40-kPa pressure cycle, where the headspace pressure decreases from 101 to 61 kPa and recovers to 101 kPa (1 atm) at 1 kPa/s. At the end of a single cycle, the air–water interface bulges upward (convex). Scale bar: cavity diameter $D$ = 200 μm

**Supplementary Note 2: Theoretical Model**

In this section, we will develop a theoretical model to predict BtP and failure cycle observed under our experimental conditions.

**Initial conditions**

The simulation tracks the gas volume inside the cavity under pressure changes and diffusion. The initial gas volume at immersion is given by $\pi D^2(h - h_{\mathrm{DR}})/4$, where $D$ and $h$ are the cavity diameter and depth, respectively (Fig. 6A in the main text). The gas pressure before immersion is $(P_{\mathrm{atm}} - P_{\mathrm{v}})$, where $P_{\mathrm{atm}} = 101.325$ kPa is the atmospheric pressure and $P_{\mathrm{v}} = 2.64$ kPa is the vapor pressure of water. Immediately after immersion, a contact angle meniscus $\theta_0$ is formed; hence, the gas volume inside the cavity changes. Consequently, the gas pressure inside the cavity after the immersion $p_{\mathrm{G,0}}$ also changes as follows:

$$p_{\mathrm{G,0}} = (P_{\mathrm{atm}} - P_{\mathrm{v}})\frac{\pi D^2(h-h_{\mathrm{DR}})/4}{V(\theta_0)}, \tag{S1}$$

where $V(\theta_0)$ is the gas volume after immersion. In general, assuming that the meniscus is pinned to the edge of the reentrant overhang with an overhang length $h_{\mathrm{DR}}$, gas volume as a function of the interfacial angle can be expressed as

$$V(\theta) = \frac{\pi D^2}{4}(h - h_{\mathrm{DR}}) + \frac{\pi D^3}{8}\frac{(2-3\sin\theta+\sin^3\theta)}{(3\cos^3\theta)}. \tag{S2}$$

Using this relation, we can probe the gas volume inside the cavity with $\theta$. This is practically easier than directly tracking the gas volume. Now, the initial external pressure can be defined as $P_{\mathrm{i}} = (P_{\mathrm{atm}} + \rho g H - P_{\mathrm{v}})$. Here, $\rho$, $g$, and $H$ are water density, gravitational acceleration, and water column thickness, respectively. Then, the initial condition can be described using the Laplace's law, $\Delta P = 2\gamma/R$, where $\Delta P$ is the pressure difference across the interface given by $P_{\mathrm{i}} - p_{\mathrm{G,0}}$, $\gamma$ is the liquid surface tension, and $R = -D\cos\theta/2$ is the curvature radius. Using the above definitions, we can write the initial condition as a function of the initial interfacial angle $\theta_0$ as

$$(P_{\mathrm{atm}} + \rho g H - P_{\mathrm{v}}) - \frac{(P_{\mathrm{atm}}-P_{\mathrm{v}})\pi D^2(h-h_{\mathrm{DR}})/4}{\frac{\pi D^2}{4}(h-h_{\mathrm{DR}})+\frac{\pi D^3}{8}\frac{(2-3\sin\theta_0+\sin^3\theta_0)}{(3\cos^3\theta_0)}} = -\frac{4\gamma\cos\theta_0}{D}. \tag{S3}$$

Here, we assumed that no hydrostatic variation occur at the curvature because the cavity size is smaller than the capillary length. Eq. (S3) can be solved numerically using the Mathematica software to obtain $\theta_0$. Subsequently, the initial gas volume can be calculated from Eq. (S2) as $V_{\mathrm{i}} = V(\theta_0)$.

## Evolution of the interfacial angle

In the experiment, the pressure in the system was increased at a specific pressure ramp rate. We simulate this by iteratively increasing the external pressure with pressure increment $P$ at time step $\mathrm{d}t$ for $N$ iterations. Using Eq. (S2) and the $P_1V_1 = P_2V_2$ relation, we can calculate the gas volume and the interfacial angle after the pressure increase $\theta_p$ as

$$\frac{P_1V_1}{P_2} = \frac{\pi D^2}{4}(h - h_{\mathrm{DR}}) + \frac{\pi D^3}{8}\frac{(2-3\sin\theta_p+\sin^3\theta_p)}{(3\cos^3\theta_p)}. \quad \text{(S4)}$$

In the first iteration, $P_1 = P_{\mathrm{i}}$, $V_1 = V_{\mathrm{i}}$, and $P_2 = P_{\mathrm{i}} + P$. Again, we can numerically solve Eq. (S4) to obtain $\theta_p$.

In addition, to describe the gas volume change due to diffusional loss, we used Fick's first law $J = -D_{\mathrm{G}}\frac{(c_0-c_\infty)}{l}$ along with the Henry's law, the ideal gas equation to obtain the following governing equation, as discussed in the main manuscript:

$$\frac{1}{A(\theta(t))}\frac{\mathrm{d}[p_{\mathrm{G}}V(\theta(t))]}{\mathrm{d}t} = -RT\frac{D_{\mathrm{G}}}{K_{\mathrm{H}}}\frac{(p_{\mathrm{G}}-s.p_{\mathrm{G},0})}{l}, \quad \text{(S5)}$$

where $p_{\mathrm{G}}$ is the current gas pressure; in this case $p_{\mathrm{G}} = P_{\mathrm{i}} + P$. For air, the values of the Henry's constant and diffusion coefficient are $K_{\mathrm{H}} = 1.30 \times 10^5$ m$^3$Pa mol$^{-1}$ and $D_{\mathrm{G}} = 2 \times 10^{-5}$ cm$^2$/s, respectively. The interfacial area $A$ can be expressed as a function of $\theta$ as follows:

$$A(\theta) = \frac{\pi D^2}{(\cos(\theta/2)+\sin(\theta/2))^2}. \quad \text{(S6)}$$

Additionally, we used $s{\sim}1$ when $l$ is in the order of $10^{-4}$ m. By substituting Eqs. (S2) and (S6) into Eq. (S5), we obtained a differential equation that describes the evolution of $\theta(t)$. The differential equation was then solved numerically for a small time step from $0 \geq t \geq \Delta t$ and using $\theta(0) = \theta_p$ as the initial value. The final $\theta$ value for this iteration was obtained by evaluating $\theta(\Delta t)$. The corresponding gas volume and pressure of this step then become the initial values for the next iteration, i.e. $P_1 = p_{\mathrm{G}}$, $V_1 = V(\theta(\Delta t))$ and $P_2 = p_{\mathrm{G}} + P$.

## Simulation procedures

We performed different simulation procedures depending on the type of experiment we wanted to replicate, such as continuous pressure ramps, cyclic pressures, and cyclic pressures with time intervals. For the continuous pressure ramp $P_{\mathrm{rate}}$, $P$ was increased by 0.1 kPa at every

time step $\Delta t$ where it was determined by $\Delta t = P/P_{\mathrm{rate}}$. The same applied to the cyclic pressures, except that, once the iteration reached $N = P_{\mathrm{max}}/P$, $P$ and $P_{\mathrm{rate}}$ became negative and the iteration continued until $p_{\mathrm{G}} = P_{\mathrm{I}}$ and completed one cycle. In addition, when the time interval $t_{\mathrm{i}}$ was used, Eq. (S5) was solved from $0 \geq t \geq t_{\mathrm{i}}$ after the completion of each cycle. These procedures were performed until the maximum contact angle $\theta_{\mathrm{max}}$ was achieved, which indicated that the interface touched the bottom of the cavity. The value $\theta_{\mathrm{max}}$ was obtained from the following geometric relation:

$$\frac{h - h_{\mathrm{DR}}}{\sin\theta_{\mathrm{max}} - 1} = \frac{D}{2\cos\theta_{\mathrm{max}}}. \qquad \text{(S7)}$$

There were some differences between the model and the experimental observations (Fig. 6). This was expected because we only considered diffusion in one dimension in our model. However, our simulation results show that our model can capture the experimental results, confirming that the governing physics is diffusion.

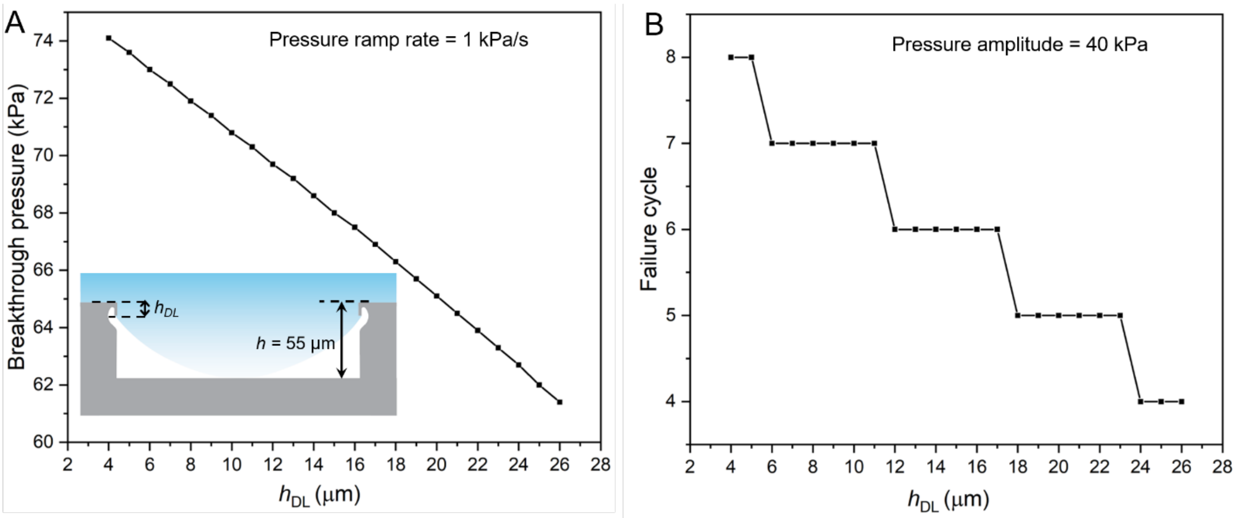


**Supplementary Figure 9:** Influence of the length of the doubly reentrant overhang on BtP and the failure cycle number. (A, B) For a fixed cavity depth ($h$ =55 μm), increasing $h_{\mathrm{DL}}$ reduces BtP and the failure cycle number. As presented in Fig. 6 and Supplementary Fig. 9(A), the longer the reentrant overhang ($h_{\mathrm{DL}}$), the lower the $\theta_{\mathrm{max}}$ and BtP. A stepwise decrease in the failure cycle number does not capture the quantitative variation in BtP shown in Supplementary Fig. 9(A). Although some $h_{\mathrm{DL}}$ values had the same number of failing cycles, they actually failed at different BtP values with small gradual differences.

Supplementary References:

1 Lv, P. Y. *et al.* Symmetric and Asymmetric Meniscus Collapse in Wetting Transition on Submerged Structured Surfaces. *Langmuir* **31**, 1248-1254, (2015).

2 Domingues, E. M., Arunachalam, S., Nauruzbayeva, J. & Mishra, H. Biomimetic coating-free surfaces for long-term entrapment of air under wetting liquids. *Nature Communications* **9**, 3606, (2018).
