# Supplementary material for: Insect-inspired breathing interfaces: investigating robustness of coating-free gas entrapping microtextured surfaces under pressure cycles
Source: Commun Eng. 2024 Jun 21;3:84. doi: 10.1038/s44172-024-00231-2 (PMC11192747; doi:10.1038/s44172-024-00231-2)
Supplement: Supplementary file 2 — Description of Additional Supplementary Files [file 44172_2024_231_MOESM2_ESM.pdf]

Description of Additional Supplementary Files

File name: Supplementary Movie S1

Description: BtP of a doubly reentrant cavity. Top view of a microtextured silica surface with a single doubly reentrant cavity immersed under a 3-mm water column and headspace air pressure increased at 1 kPa/s until the interface touched the cavity floor. Movie playback speed: 5× faster

File name: Supplementary Movie S2

Description: Monotonically depleting air pocket inside the doubly reentrant cavity under cyclic pressure (no interval). Top view of a microtextured silica surface with a single doubly reentrant cavity immersed under a 3-mm water column and 40-kPa-amplitude cyclic pressure applied continuously at 1 kP/s. Movie playback speed: 48× faster

File name: Supplementary Movie S3

Description: Indefinitely stable air pocket inside the doubly reentrant cavity under cyclic pressure with interval time. Top view of a microtextured silica surface with a single doubly reentrant cavity immersed under a 3-mm water column. A 40-kPa-amplitude cyclic pressure was applied at 1 kPa/s, and the interval between cycles varied between 4, 5, 6, and 8 min. An indefinitely stable air pocket was observed at 6- and 8-min intervals. Movie playback speed: 1320× faster

File name: Supplementary Movie S4

Description: Stability of the air pocket inside the doubly reentrant cavity subjected to a cyclic pressure pattern similar to human breathing. Top view of a microtextured silica surface with a single doubly reentrant cavity immersed under 6- and 2-mm water columns. The breathe-in pressure was set to 8 kPa, breathe-out pressure was set to 3.5 kPa, breathing duration (in/out) was set to 1.5 s, and time interval between each cycle was 0 s. An indefinitely stable air pocket was observed for a 2-mm water column. Movie playback speed: 2200× faster

File name: Supplementary Movie S5

Description: Air pocket growing outside the doubly reentrant cavity under negative pressure cycles (no interval). Top view of a microtextured silica surface with a single doubly reentrant cavity immersed under a 3-mm water column and a 40-kPa amplitude cyclic pressure applied continuously at 1 kP/s using vacuum suction. Movie playback speed: 2200× faster

File name: Supplementary Movie S6

Description: Stable interface after bubble departure from the doubly reentrant cavity due to mechanical vibration. The air pocket grew outside the doubly reentrant cavity because of the 40- kPa-amplitude cyclic pressure applied using vacuum suction. After the departure of a significant portion of the bubble because of mechanical vibration, DRCs recovered to their initial Cassie state. Movie playback speed: 1×

File name: Supplementary Movie S7

Description: Air pocket growing outside the doubly reentrant cavity under positive–negative pressure cycles (no interval). DRCs immersed in 3-mm-thick water columns were subjected to alternating positive–negative pressure cycles of amplitude ±40 kPa (at a ramp rate of ±1 kPa/s and ti = 0) via a combination of positive air pressure and vacuum suction. The trapped air bubble grew in size over time. Movie playback speed: 4200× faster
